# A Medial Subvastus Approach for Lateral Unicompartmental Knee Arthroplasty: Technique Description and Early Outcome Results

**DOI:** 10.1016/j.artd.2021.04.013

**Published:** 2021-06-18

**Authors:** Robert M. Fuller, Daniel I. Wicker, Grace W. Getman, Katherine S. Christensen, Christian P. Christensen

**Affiliations:** a Middlebury College, Middlebury, VT, USA; b Bluegrass Orthopaedics, Lexington, KY, USA; c Vanderbilt University, Nashville, TN, USA

**Keywords:** Unicompartmental, Lateral, Subvastus, Knee, Arthroplasty

## Abstract

**Background:**

Unicompartmental knee arthroplasty (UKA) treats arthritis involving only one compartment of the knee. Lateral UKA is mainly performed through medial parapatellar or lateral parapatellar approaches to the knee. This technique article introduces a medial subvastus approach to lateral UKA, discusses the clinical rationale behind its use, and offers a preliminary retrospective study on short-term outcomes of lateral UKAs using the lateral vs medial subvastus approaches.

**Methods:**

A description of the medial subvastus approach is included. In addition, we reviewed 32 and 30 lateral UKAs performed using the lateral and medial subvastus approaches, respectively. Minimum follow-up duration was 1 year. Knee injury and osteoarthritis outcome score for joint replacement (KOOS, JR) knee scores were used for comparison.

**Results:**

Age and body mass index were similar between the 2 cohorts. Mean KOOS, JR. scores for the subvastus approach group were significantly higher than those for the lateral approach group at 81.41 ± 2.0 for medial subvastus and 74.19 ± 2.9 for lateral (*P* = .02). One deep infection and 2 revision total knee arthroplasties occurred in the lateral approach group. Neither occurred in the subvastus group. The mean follow-up duration was significantly longer for the lateral approach group than that for the subvastus group at 749 vs 410 days (*P* < .001). Literature on time-dependence of patient-reported outcomes supports usage of the data, despite follow-up discrepancies.

**Conclusions:**

A subvastus approach for lateral UKA may offer improved visualization, easier conversion to total knee arthroplasty, and faster recovery, based on clinical observation. Preliminary results suggest improved short-term knee scores compared to a lateral approach.

## Introduction

Total knee arthroplasty is a very common and successful operative intervention to treat arthritis of the knee. Despite its success, numerous studies have demonstrated a 20% rate of patient dissatisfaction with this operation [[1], [2], [3], [4]]. As such, many surgeons have turned to unicompartmental knee replacement (UKA) to treat patients with arthritis involving only one of the 3 compartments of the knee.

Lateral compartment degenerative joint disease and lateral partial knee replacement comprise less than 1% of all knee replacements performed in North America [5]. Lateral unicompartmental knee arthroplasty has traditionally been performed through both medial parapatellar and lateral parapatellar approaches to the knee. Significant controversy exists regarding the optimal approach for lateral UKA [6]. While many surgeons are comfortable with the medial parapatellar approach, which is commonly used in total knee arthroplasty, this approach is less popular for lateral UKAs because of problems with patellofemoral articulation, damage to the quadriceps mechanism, and risk of injury to the medial meniscus [7,8]. Lateral parapatellar arthrotomies are currently more popular, as they provide direct access to the lateral compartment, but present greater difficulty with conversion to total knee arthroplasty, both during the index UKA procedure and on revision [8].

A medial subvastus approach, sometimes used in total knee arthroplasty, was considered as an alternative to each of these options for lateral partial knee replacement as it is a quadricep-sparing approach with a lower impact on patellofemoral articulation, while offering improved exposure and simpler conversion to total knee arthroplasty in the clinical opinion of one of this article’s authors, a fellowship-trained adult reconstructive surgeon [9]. Meta-analyses of total knee arthroplasty using the medial subvastus approach vs medial parapatellar arthrotomies also suggest improved recovery times, decreased blood loss, and better 1-year patient-reported outcome [10]. The senior author switched from using lateral parapatellar approach to the novel medial subvastus technique in a high-volume joint replacement practice based off of his own clinical considerations. This technique article aims to explain the medial subvastus approach for lateral unicompartmental knee arthroplasty, as well as to offer a retrospective, exploratory study comparing short-term patient-reported outcomes of lateral UKAs performed through the medial subvastus and lateral approaches.

### Surgical technique for subvastus approach

**Figure 1:**
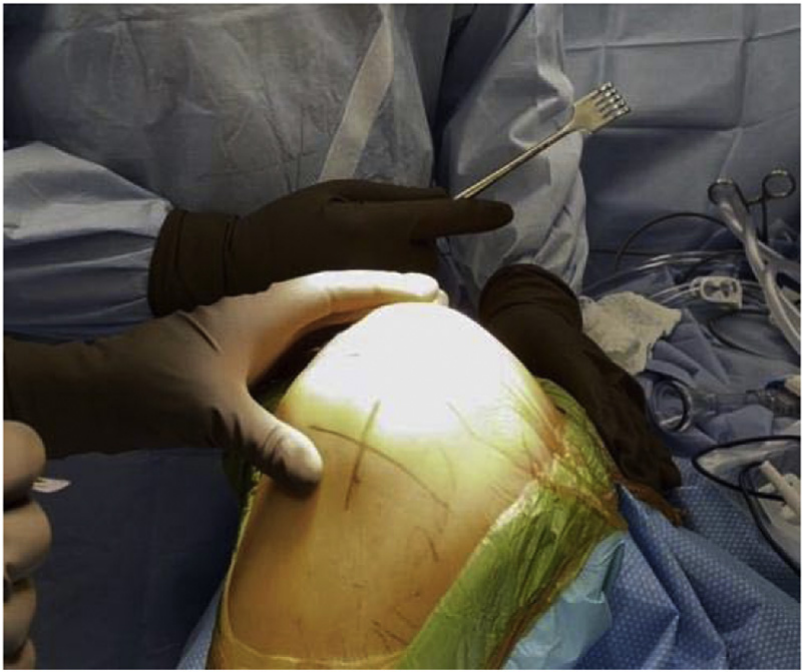
Knee positioning.

**Figure 2:**
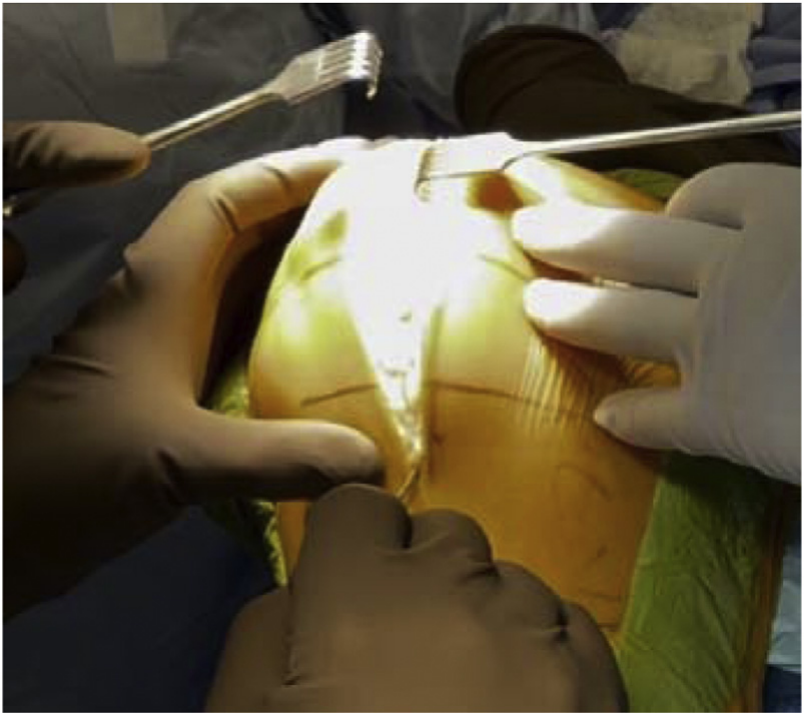
Midline incision.

**Figure 3:**
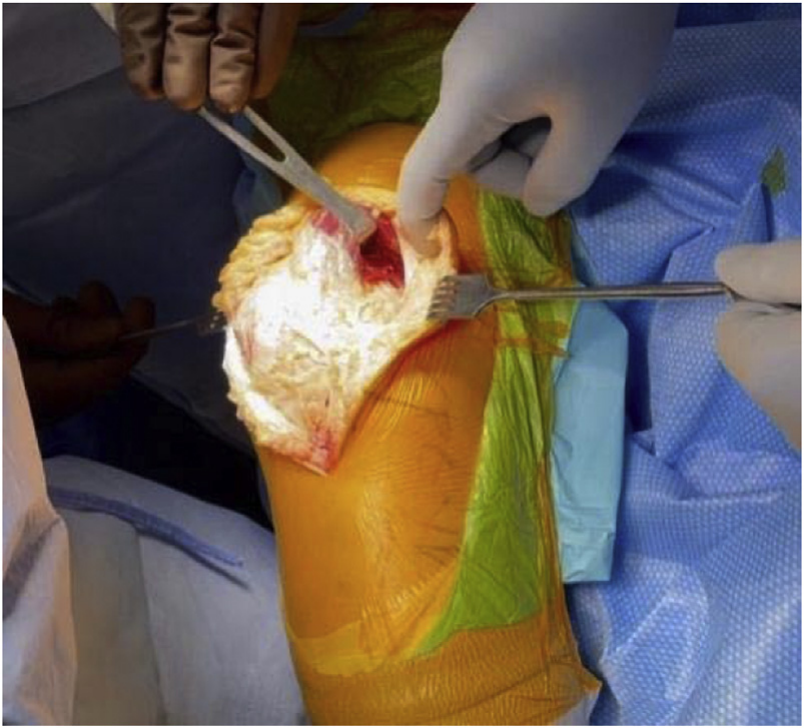
Elevation of VMO musculature.

**Figure 4:**
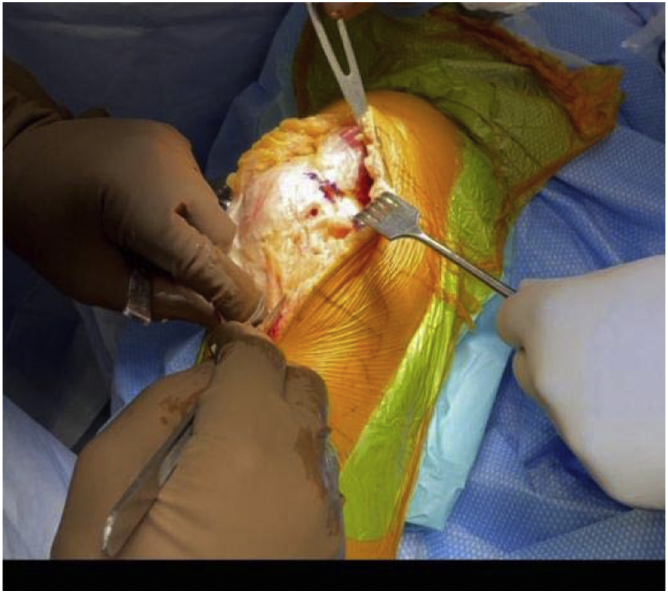
Distal capsular incision.

**Figure 5:**
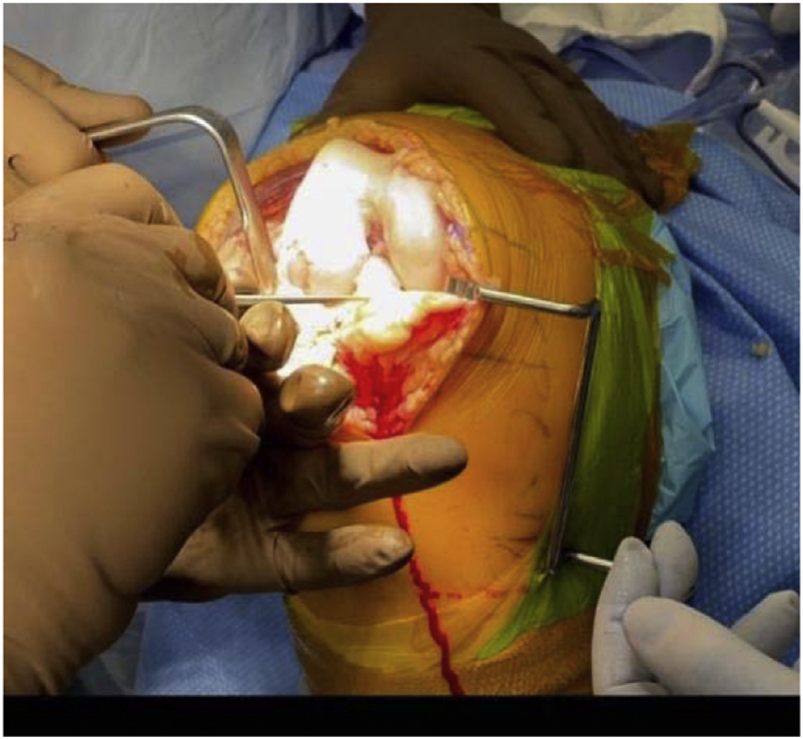
Inspection of knee compartments.

**Figure 6:**
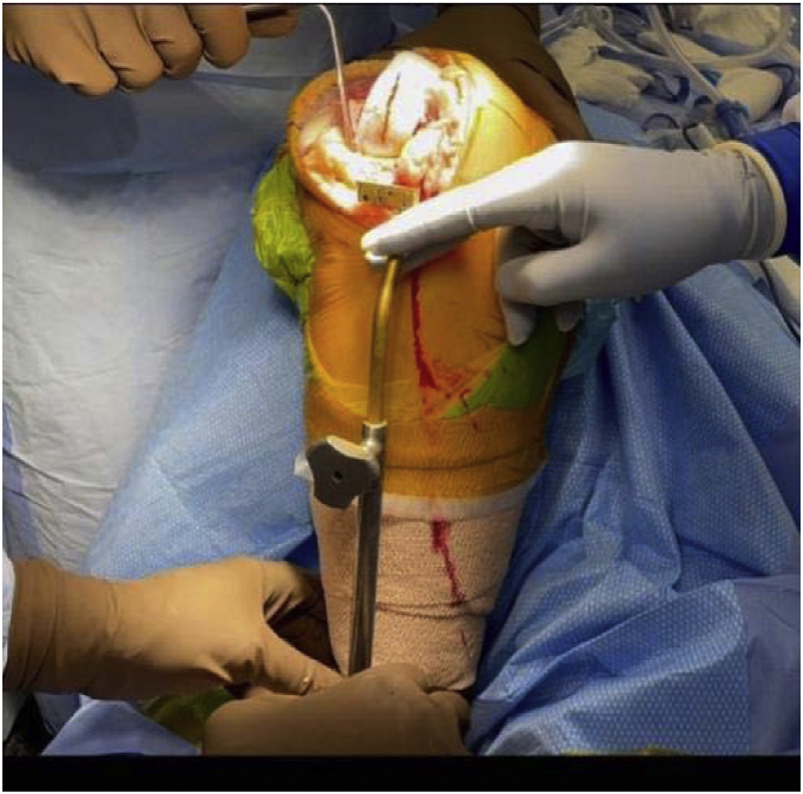
External tibial alignment guide.

In March 2018, a single surgeon began using the subvastus approach for lateral unicompartmental knee arthroplasty. In all cases, standard anesthesia, skin preparation, and draping for total knee arthroplasty were used (Fig. 1). A midline incision was made over a flexed knee (Fig. 2). Proximal dissection was performed over the medial aspect of the quadriceps extending approximately 10 cm above the proximal pole of the patella. Blunt dissection was performed over the medial aspect of the vastus medialis obliquus (VMO) musculature, and digital elevation was used to separate the medial border of the VMO from the intermuscular septum. An Army-Navy Retractor was placed on the medial aspect of the VMO, exposing the proximal knee capsule with the knee flexed approximately 45° (Fig. 3). A gentle incision was made into the joint capsule medial to the patella extending distally along the medial aspect of the patellar tendon. Care was taken not to penetrate into the medial femoral condyle cartilage or the anterior horn of the medial meniscus. The capsular incision extended proximally from the patella underneath the vastus medialis. At this point, the knee was extended fully to complete the capsular incision. Dissection distally proceeded from the medial aspect of the patellar tendon, posterior to the tendon but anterior to the fat pad to avoid any injury to the anterior horn of the medial meniscus or the intermeniscal ligament (Fig. 4). The patella was then everted, and the knee was gently flexed to 120°. A 90-degree Hohman retractor was placed lateral to the midsection of the lateral tibial plateau. The anterior cruciate ligament and the medial and patellofemoral compartments were carefully inspected, including the entire anterior half of the medial meniscus that can be seen with deep knee flexion (Fig. 5). Partial removal of the patellar fat pad was performed to optimize visualization of the entire lateral compartment. After the patient was confirmed to be a suitable candidate for lateral unicompartmental knee arthroplasty, the lateral meniscus remnant was excised. Careful, sharp separation of the anterior and lateral aspects of the lateral tibial plateau from the surrounding soft tissue was performed to facilitate easier bone removal after lateral tibial plateau osteotomy. At this point, an external tibial alignment guide was used to resect the lateral tibial plateau and remove approximately 2-4 mm of bone depending on bone loss (Fig. 6). Lateral UKA was then performed routinely per manufacturer’s instruction.

The lateral parapatellar approach to lateral unicompartmental knee arthroplasty has been thoroughly described in existing literature and should be familiar to surgeons performing lateral UKA. For reference, we recommend the following article—“Lateral Unicompartmental Knee Arthroplasty Through a Lateral Parapatellar Approach Has High Early Survivorship”—which contains an excellent description of the lateral parapatellar technique with pictures of exposure [11].

## Material and Methods

This retrospective study compares 32 consecutive lateral unicompartmental knee arthroplasties in 31 patients performed through a lateral approach with 30 consecutive lateral partial knee replacements in 30 patients performed using a medial subvastus approach. All surgeries were performed by a single, fellowship-trained adult reconstruction surgeon. During the first cohort period, the surgeon also performed 706 total knee arthroplasties and 633 medial UKAs. During the second cohort period, the same surgeon performed 521 TKAs and 264 medial UKAs. All patients were carefully evaluated preoperatively with a thorough physical and radiographic examination. Preoperative knee radiographs included a posterior-anterior flexed view, a lateral radiograph, a merchants’ view, and a stress varus radiograph that demonstrated correction of the valgus deformity without significant medial compression. The first cohort using the lateral approach with a leg holder was performed between April 2016 and March 2018. The second cohort using the subvastus approach on a standard operating room table began in March 2018 and ended in April 2019. The Zimmer Biomet Fixed Lateral Oxford implant (Warsaw, IN) was used in all cases, regardless of approach.

Descriptive statistics were used to summarize the demographic data of the 2 cohorts. Age, gender, and body mass index were the demographic characters compared between the 2 cohorts using Welch’s t-tests for numerical data and Pearson’s chi-squared tests for categorical data. Follow-up time between the 2 cohorts was also compared for statistical significance using a Welch’s t-test. Minimum follow-up duration was 1 year for both cohorts. Knee injury and osteoarthritis outcome score for joint replacement (KOOS, JR) knee scores were obtained via phone interview at a minimum of 1 year postoperatively (Table 1) [12]. Three patients from the lateral approach cohort could not be reached for follow-up out of a series of 35 individuals. These patients were excluded from analysis. No patients were excluded from the medial subvastus cohort. A Welch’s t-test was used to analyze differences in means and distributions between the lateral and medial subvastus approach cohorts and were used to compare the 2 cohorts. An alpha-level of *P* < .05 was considered statistically significant for all statistical tests. Analyses were performed by a biostatistician using R software.

**Table 1:** KOOS, JR Survey items.

Knee injury and osteoarthristis outcome score for joint replacement (KOOS, JR)
Stiffness
1. How severe is your knee stiffness after first wakening in the morning?
Pain: What amount of knee pain have you experienced in the last week during the following activities?
2. Twisting/pivoting on your knee
3. Straightening knee fully
4. Going up or down stairs
5. Standing upright
Function, daily living: The following questions concern your physical function. By this we mean your ability to move around and to look after yourself. For each of the following activities please indicate the degree of difficulty you have experienced in the last week due to your knee.
6. Rising from sitting
7. Bending to floor/pick up an object

## Results

**Figure 7:**
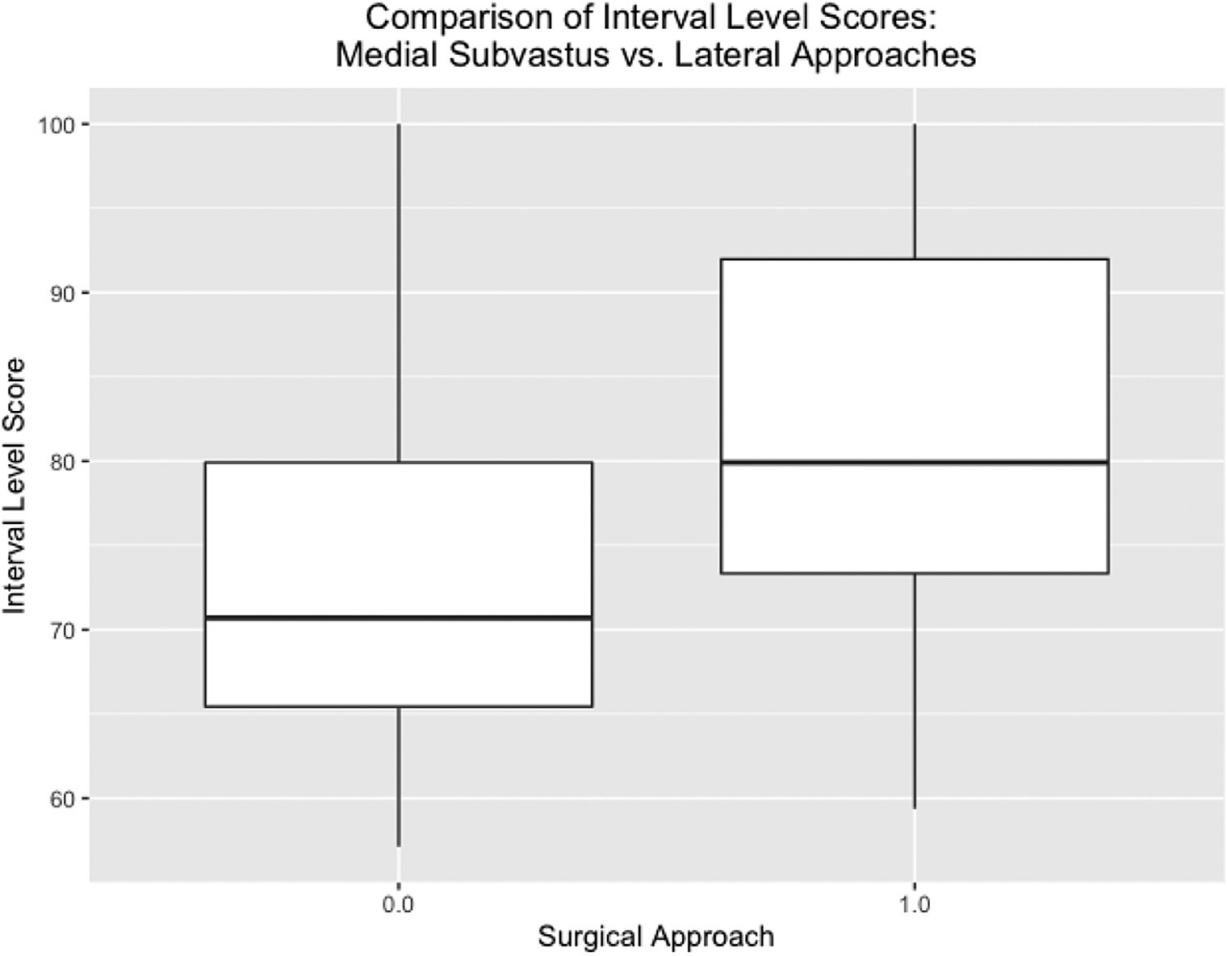
Comparison of interval level scores: medial subvastus vs lateral approaches.

The mean follow-up time for the lateral approach group was significantly longer than that for the subvastus approach group (749 days vs 410 days, *P* < .001). Patient age, body mass index, and gender were similar between the 2 cohorts (Table 2). KOOS, JR knee scores were statistically superior for the subvastus approach group. The mean score for the subvastus approach group was 81.41 ± 2.0, while the mean score for the lateral approach group was 74.19 ± 2.9 (*P* = .02) (Fig. 7). Medial subvastus patients reported superior outcomes for every item of the KOOS, JR survey. Statistically significant differences were found between the 2 cohorts for questions 2, 4, 5, and 6 of the KOOS, JR surveys (Table 3). There was also a statistically significant difference in tourniquet time between the 2 cohorts, with the average tourniquet time at 63.2 minutes and 57.7 minutes in the lateral approach and subvastus approach cohorts, respectively (*P* < .001). In addition, there was one deep infection in the lateral approach group and 2 revisions to total knee arthroplasty. One revision was due to medial femoral compartment degeneration, and the other due to the aforementioned infection. While neither of these complications merited exclusion from the study, one of the 2 revision patients (with the infection) was unable to be reached for follow-up and subsequently excluded. There were no infections or revisions in the subvastus approach group. There was one knee arthroscopy in the lateral approach group for a medial meniscus tear.

**Table 2:** Demographic data organized by surgical approach.^a^

Case number	Lateral	Medial subvastus	*P* value
	32	30	
Age (y)	69.2	71.9	.254
Body mass index	30.1	28.5	.301
Gender (%female)	86.7	87.1	.718
Follow-up time (d)	749	410	8.04E-15

a Demographic data stratified by approach shows no statistically significant differences between the 2 cohorts. Follow-up time of the KOOS JR surveys, however, show a statistically significant difference in days after surgery collected.

**Table 3:** Individual KOOS, JR item response averages by surgical approach.

KOOS, JR question	Lateral approach, mean score	Medial subvastus, approach mean score	Significance value
1	0.724137931	0.4838709677	0.1663443959
2	0.9310344828	0.4838709677	**0.01854680848**
3	0.6896551724	0.6451612903	0.7952077631
4	0.8620689655	0.4838709677	**0.03673219482**
5	0.9655172414	0.4838709677	**0.0130184671**
6	0.7931034483	0.3225806452	**0.004098274723**
7	0.724137931	0.5483870968	0.3438517878

Values in bold are statistically significant values (P < .5).

## Discussion

This study supplements clinical observations on the operational and recovery-related outcomes of a novel medial subvastus approach to lateral UKA with quantitative patient-reported outcome data. The surgeon-author began using a subvastus approach for numerous reasons. Based on surgical experience, when compared to a lateral approach, the medial subvastus method offers superior medial compartment visualization and assessment of the anterior cruciate ligament. It allows easier and more thorough intraoperative conversion to total knee arthroplasty as well as easier postoperative irrigation and debridement for infection should either be necessary. At the same time, the medial subvastus approach allows patients a rapid rehabilitation without compromising patellar tracking, unlike the medial parapatellar approach, which diminishes the medial pull of the VMO. This lower postoperative VMO pull potentially elevates lateral facet pressure on the patella in a patient population predisposed to lateral sided patellofemoral degeneration [13]. Finally, the medial subvastus approach appears to allow for improved use of an intramedullary rod into the femoral canal, which can improve femoral implant position and subsequently reduce the risk of revision.

This study’s results suggest that the medial subvastus approach may provide meaningful improvements in short-term patient-reported outcomes when compared to a lateral approach. Patients who underwent lateral UKA via medial subvastus approach reported significantly greater overall KOOS, JR scores on follow-up, driven by improvements in pain and functionality. Specifically, significant differences were found for patient experiences with twisting or pivoting the knee, going up or down stairs, standing upright, and rising from the seated position. Moreover, patients in the medial subvastus group had no revisions or infections, unlike the 2 revisions necessary for the lateral group, one of which was to address an infection. While meaningful conclusions cannot be drawn for complication rates, these observations also warrant further investigation into the utility of the medial subvastus approach as an alternative to lateral and medial parapatellar approaches. The statistically significant difference in mean tourniquet time between the 2 cohorts (5.5 minutes, *P* < .001) should be noted and further investigated, as it may indicate potential advantages conferred by the subvastus approach.

This study has a few deficiencies that the authors would like to address. First, we recognize that the small sample size (n = 62 patients, split between 2 groups) leaves the study relatively underpowered. The rarity of lateral unicompartmental arthroplasty makes it difficult to compile large patient populations [[14], [15], [16], [17]]. Ideally, a follow-up publication with more data will eventually supplement this technique article to provide a fuller picture of patient-outcome differences. However, despite the small sample, the data offer strong preliminary evidence that a medial subvastus approach to lateral UKA may warrant further investigation as a suitable alternative to lateral parapatellar or medial parapatellar arthrotomies.

We recognize that the follow-up time in the lateral approach group is significantly longer at approximately 24 months vs a mean follow-up time of approximately 24 months in the medial subvastus group. This leaves the possibility that the results of the KOOS, JR knee scores for the subvastus approach group may decline with longer follow-up time. While this drawback must be noted, we believe that the data presented in this study will still be useful for 2 reasons. First, analysis of the time-dependence of patient-reported outcome measures in knee arthroplasty consistently demonstrates that outcomes reliably improve, rather than decline, through the first 2 years after surgery [11,18,19]. These observations hold not just for lateral unicompartmental knee arthroplasty, but medial UKA and total knee arthroplasty as well. Therefore, it is reasonable to expect that the trend toward better outcomes in the medial subvastus data may actually widen over time. Second, the purpose of this study is not to settle a question, but rather to pose one. As noted previously, the switch to the novel approach was spurred not by academic considerations, but rather by clinical ones. It is our hope that the publication of these findings will spur further research into the relative merit of a medial subvastus approach. However, the results of this study should be interpreted with caution and clinical judgment until more literature can confirm these findings.

## Conclusions

This technique article offers both a technical description of the medial subvastus approach for lateral unicompartmental knee arthroplasty and the clinical rationale behind its use as an alternative to medial or lateral parapatellar approaches. It also introduces a preliminary retrospective study suggesting that a subvastus approach for lateral unicompartmental knee arthroplasty may offer improved short-term knee outcomes and should be further investigated as a potential alternative to the lateral parapatellar and medial parapatellar approaches.

## Conflicts of interest

The authors declare that they have no known competing financial interests or personal relationships that could have appeared to influence the work reported in this article.

## Appendix A Supplementary data

Conflict of Interest Statement for Christensen

Conflict of Interest Statement for Getman

Conflict of Interest Statement for Christensen

Conflict of Interest Statement for Wicker

Conflict of Interest Statement for Fuller
